# Therapeutic Potentials of Virtual Blue Spaces: A Study on the Physiological and Psychological Health Benefits of Virtual Waterscapes

**DOI:** 10.3390/healthcare13111353

**Published:** 2025-06-05

**Authors:** Su-Hsin Lee, Yi-Chien Chu, Li-Wen Wang, Shu-Chen Tsai

**Affiliations:** 1 Department of Geography, National Taiwan Normal University, Taipei 10644, Taiwan; shlee@ntnu.edu.tw (S.-H.L.); joyce2008i@gmail.com (Y.-C.C.); 2 College of Arts and Design, Jimei University, Xiamen 361021, China; tsaishuchen@jmu.edu.cn

**Keywords:** virtual reality (VR), emotional regulation, systolic blood pressure, retirees, digital health strategies, psychophysiological responses, virtual nature exposure

## Abstract

**Background:** Physical and mental health issues are increasingly becoming a global focus of attention, and telemedicine is widely attracting academic interest. Objectives: This exploratory study aimed to investigate the therapeutic potential of immersive virtual blue spaces for individuals with distinct lifestyle backgrounds—specifically, office workers and retirees. The research explores how different virtual waterscapes influence emotional and physiological states in populations with varying stress profiles and life rhythms. **Methods**: A mixed-methods design was employed, combining quantitative measurements with qualitative interviews. In September 2023, forty participants (20 office workers and 20 retirees) from Hualien, Taiwan, were exposed to 360° VR simulations of three blue environments: a forest stream, a forest waterfall, and a beach scene. Pre- and post-session assessments included physiological indicators (blood pressure and heart rate) and emotional states measured using the Profile of Mood States (POMS) scale. **Results**: Significant physiological relaxation was observed among retirees. Office workers demonstrated greater emotional improvements, with noticeable variation depending on the type of virtual environment. Comparative analysis highlighted the stream landscape’s unique benefit for reducing depression and enhancing positive mood states. Thematic findings from post-session interviews further indicated that emotional responses were moderated by individual background and prior emotional experiences. **Conclusions**: These findings underscore the short-term therapeutic potential of virtual blue spaces for diverse user groups and reveal the influence of personal context on their effectiveness. The study supports the integration of VR-based nature exposure into personalized digital healthcare interventions and offers a foundation for future development in immersive therapeutic technologies.

## 1. Introduction

Recently, mental health issues have impacted around 970 million individuals globally, representing approximately 12.5% of the world’s population [1]. The rising prevalence of anxiety, depression, and other mental health challenges has fueled the demand for wellness tourism. The COVID-19 pandemic has further amplified the need for nature-based wellness tourism, establishing it as a new normal market in the tourism sector’s recovery [2]. Health tourism that utilizes natural settings for therapeutic benefits, such as forest therapy tourism, is becoming increasingly popular. This type of tourism promotes both mental and physical health by providing experiences that involve engaging with nature. It effectively reduces depression and anxiety while enhancing overall life satisfaction [3,4,5].

Natural settings, including green, blue, and yellow spaces [6,7], have shown considerable benefits for physical and mental health. The “Biophilia Hypothesis” suggests an evolutionary connection between humans and nature, benefiting mental health [8]. Forest healing, known as nature therapy in North America and Shinrin-yoku in Japan, harnesses natural environments’ restorative powers [8,9]. Tourism researchers are exploring sensory experiences in forest recreation and outdoor tourism’s potential for natural therapies [10]. Research shows that nature reduces stress, anxiety, and depression while enhancing emotional and cognitive functions [11,12], and also reduces blood pressure [13,14,15].

Investing in research to explore the psychological advantages of various natural settings is worthwhile [16]. Initial findings indicate that environments such as watersheds, which include lakes, rivers, streams, and wetlands, as well as riparian zones, are beneficial for mental and psychological health [17]. There is a positive link between visiting rivers and enhanced psychological well-being [18]. Among blue spaces, coastal areas are regarded as therapeutic landscapes [19]. When people seek relaxation or recovery, they tend to prefer blue spaces over green ones [20]. Compared with green spaces, such as urban parks and forests, there is relatively limited research on the potential health benefits of exposure to blue spaces [21]. Nature supports both physical and mental health in older adults, with blue spaces offering more therapeutic benefits for mental recovery and mental health in this demographic [22]. Research on healing waterscapes that clarifies the characteristics and functional mechanisms of waterscape qualities, such as freshness, mobility, sound, and cultural value, is still lacking [17,23]. However, most retirees are also elderly and may be limited by their mobility and unable to experience the therapeutic benefits of the physical properties of water features on site. If the therapeutic potential of VR is scientifically recognized and it breaks through spatial barriers, it can become a part of digital medicine [24]. The latest research indicates that telemedicine can improve patients’ access to healthcare, especially in low- and middle-income countries [25]. Similarly, it can also greatly benefit remote villages where there is a large urban–rural gap [26]. Despite preliminary evidence that exposure to blue space can benefit physical and mental health [27,28], research on digital applications for older populations remains limited [29].

The underlying mechanisms, optimal exposure parameters, and effective integration into digital health systems, such as VR-based interventions, require further investigation. This gap restricts the clinical translation of VR-based interventions into geriatric care or home-based therapeutic settings [29]. Closing this gap is crucial for developing evidence-based strategies to enhance retirees’ well-being through accessible, technology-mediated nature experiences. VR provides an innovative way to engage with destinations or attractions through immersive simulations, particularly in delivering the therapeutic effects of natural settings [30]. Additionally, destinations can leverage VR as an alternative for visitors who cannot physically access the site, such as those with mobility limitations.

Virtual exposure to natural settings can offer restorative benefits, as evidenced by research indicating that VR nature experiences can enhance mood and alleviate anxiety [31,32]. Engaging with VR forest scenes via head-mounted displays (HMDs) has been linked to mood improvements [33,34] and stress relief [35]. The restorative impact is further amplified by multisensory stimulation in VR. Ojala et al. found that brief exposure to audiovisual forest or water scenes provided greater recovery benefits than auditory stimulation or control groups alone [36]. When using an HMD for static viewing, there was no significant difference between 360° panoramic images and computer-generated environments in terms of presence, anxiety reduction, and emotional state improvement, although 360° true panoramas were more practical and cost-effective [37].

The literature review highlighted that most restorative virtual nature studies predominantly involved healthy young individuals, particularly students and office workers. Hence, future research should focus on diverse populations, including retirees and clinical patients, to broaden the generalizability and therapeutic potential [38]. Additionally, the optimal types of virtual environments and the influence of individual differences on psychological and physiological outcomes should be clarified [30].

Research is still lacking on the health-promoting benefits of various VR natural environments for individuals from different socioeconomic backgrounds. Therefore, this study targeted the subjects where current research results are most lacking: office workers and retirees. By comparing extreme urban landscapes with natural landscapes, this study explored the physical and psychological differences between two subjects with very different life rhythms under different environmental experiences.

Consequently, this study utilized a 360° VR simulation featuring three types of waterscapes—forest-based waterfalls, forest-based streams, and beaches—to investigate the psychological and physiological health benefits for office workers and retirees. This study aimed to (1) assess the impact of VR waterscapes on the physiological and psychological well-being of office workers and retirees; (2) compare the physiological and psychological effects of the three VR waterscapes; and (3) explore the personal emotional experience factors that affect the mental health benefits gained from VR waterscape exposure.

## 2. Materials and Methods

### 2.1. Research Design, Hypotheses, Materials, and Procedure

#### 2.1.1. Background of the Study Area

Although studies in Denmark, South Korea, Japan, and New Zealand have shown that a good urban environment (such as a city with cultural and historical heritage) can be a healthy landscape [6,39,40,41], densely populated urban environments remain a source of stress for most people, whether in Sweden in the northern hemisphere or New Zealand in the southern hemisphere [42,43,44]. Therefore, in recent years, major Asian countries, including China [7,45], South Korea [3,40,46,47,48,49,50], Japan [6,51,52], and Taiwan [44,53,54], have attached great importance to the healing research of natural landscapes, showing the urgency of Asian cities to solve the health problems caused by dense populations. Recent findings suggest that exposure to urban areas generally leads to higher stress levels compared with natural landscapes, with physiological indicators such as heart rate and galvanic skin response indicating increased stress in urban conditions [42]. The main factors are air pollution and high noise levels in urban environments [55,56]. Therefore, the city as a stressor and the therapeutic benefit of natural landscapes are important directions for selecting research sites.

In Asia, Taiwan’s healing-related research and policy strategies are mainly learned from Japan, China, and South Korea. The main reason is the high density of Asian cities and similar cultural backgrounds. Nevertheless, Taiwan’s geographical scale is particularly small compared with other countries, so it remains unique. Therefore, there is still much room for research projects to be explored. Taiwan is suitable as a research object, especially in the context of high-pressure employment and aging [57], and the research results can immediately reflect society’s needs.

Hualien County is the largest county in Taiwan and is located in the mountainous area of eastern Taiwan. Only 7% of Hualien County’s land is habitable plains, 87% is mountainous, and the remaining 6% is rivers. Hualien County is located adjacent to the Pacific Ocean and is famous for its stunning cliff scenery on the east coast. Hualien County attracts domestic and foreign tourists with its beautiful scenery. In 2023, the number of tourists reached more than 16.98 million, setting a record in the history of Hualien County. However, Hualien County is the second least populous county on the island of Taiwan, with a population of 314,000 in 2025 and a population density of only 68 people per square kilometer. As of the end of October 2024, the elderly population in Hualien County was 64,608, and the proportion of the elderly population was 20.47%, which is higher than the proportion of 19.03% in Taiwan [58].

**Figure 1:**
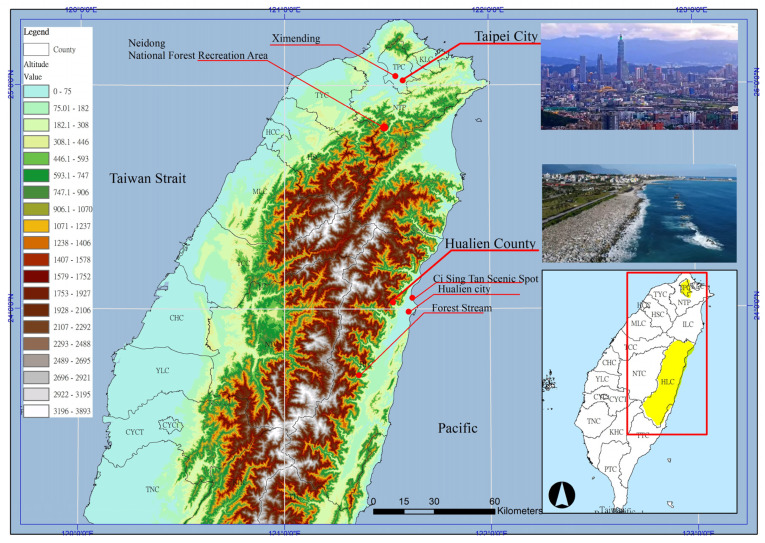
Study area.

In comparison, Taipei, Taiwan’s largest city, has a population of 2.46 million and a population density of 9070 people per square kilometer. As of June 2024, the proportion of the retiree population in Taipei City reached 22.5% [59]. The Taipei metropolitan area (TMA) is a medium-sized but densely populated metropolitan area in Taiwan [60]. Therefore, the two regions have different socioeconomic, demographic, and geographical characteristics. Hualien and Taipei represent the two ends of Taiwan’s natural and urban landscapes (Figure 1).

#### 2.1.2. Study Framework

A purposive sampling strategy was adopted to include participants representing two distinct lifestyle groups: office workers (typically experiencing higher daily stress and fast-paced routines) and retirees (with lower stress and slower lifestyles). This sampling method was selected based on this study’s aim to compare psychophysiological responses to VR waterscapes across contrasting social roles and life rhythms. All 40 participants were residents of Hualien City or nearby districts, had normal or corrected-to-normal vision, were in stable health, and could complete the immersive VR sessions.

The Ethics Review Committee at National Taiwan Normal University approved this study (202306HM002), and participants were briefed on the research procedures before it began. This research utilized a pre-test–post-test design to investigate how various VR waterscapes impacted participants’ physiological and psychological health.

This study did not use skin conductance levels as used in previous studies [42]; instead, visual and auditory cues were used as the main input sources. This study used photos and sounds of the city as stress-inducing sources. This study was conducted as an exploratory case study. The exploratory case study method can provide a deep and broad understanding of the research case and has the advantage of observing social phenomena and real life simultaneously. Because the nature of an exploratory case study is open, the first step of this study was to collect data, conduct observations, and then propose research hypotheses [61,62].

**Figure 2:**
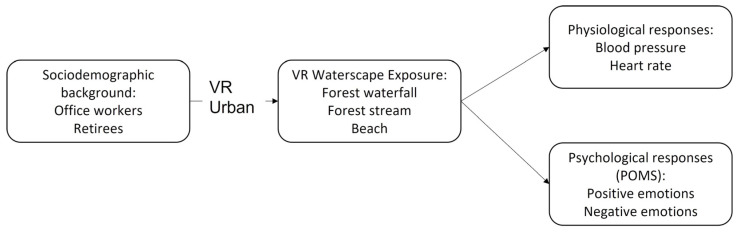
Research framework.

Participants then experienced three types of VR waterscapes: a forest waterfall, a forest stream, and a beach. Their physiological and psychological responses were measured in both the pre-test and post-test phases, with at least four hours between trials to reduce carryover effects. After the experiment, qualitative interviews were conducted to collect participants’ feedback on the VR experience (Figure 2).

#### 2.1.3. Study Hypothesis

**Figure 3:**
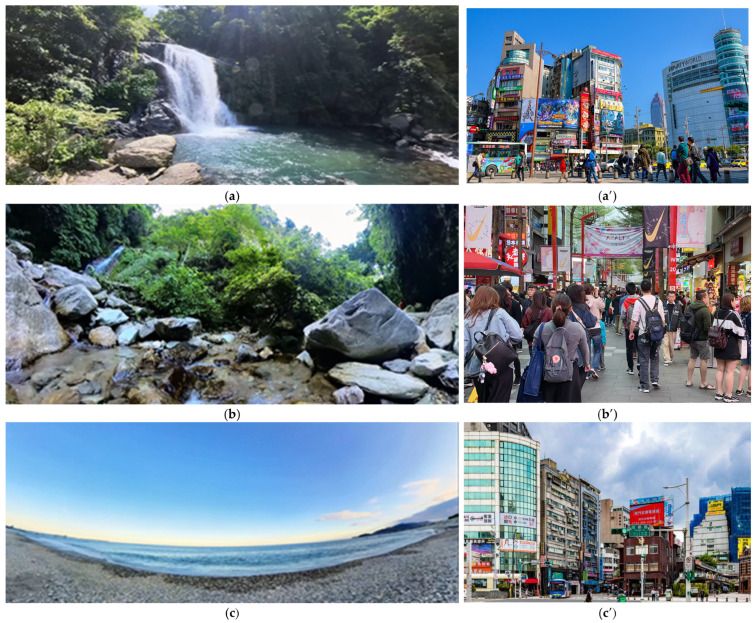
Sampled and recorded panoramic videos of three distinct waterscapes in Taiwan: (**a**) forest waterfall in Neidong National Forest Recreation Area in Taipei County, (**b**) forest stream in the suburban mountains of Hualien City, (**c**) Ci Sing Tan Scenic Spot, beach of Hualien City. (**a**’), (**b**’), and (**c**’) are the different street scenes of Ximending, the busiest business district in Taipei City.

This study included stress as an inducer. The definition of stress depends on the research object. For example, in a study focusing on students, a math quiz is used as the source of stress [63]. However, this study used urban scenery as a source of stress to compare the differences between office workers and retirees, because Taiwanese people generally imagine wanting to escape the city and return to their hometown after retirement. Yu et al. [57] used a natural landscape to contrast with an urban landscape. The experimental samples gained recovery from the natural landscape but not from the urban landscape. This study further tested whether an urban landscape itself is the source of stress. This study used the natural waterscape of Hualien as the stress relaxation site (Figure 3a–c) and a bustling urban scene as a stress-inducing hypothetical factor for participants. The urban video, chosen from Ximending (Figure 3a’–c’), a popular shopping area in Taipei City, Taiwan [30], featured elements typical of city life, including large crowds, traffic noise, and closely packed buildings.

**Hypothesis** **1.** *The effects of VR waterscapes on physical and mental well-being vary between office workers and retirees*.

**Hypothesis** **2a.** *Among office workers, the health advantages, both physiological and psychological, of exposure to different VR waterscapes differ*.

**Hypothesis** **2b.** *Among retirees, the health advantages, both physiological and psychological, of exposure to different VR waterscapes differ*.

**Hypothesis** **3.** *The mental health benefits derived from VR waterscape exposure are moderated by individual emotional experience factors*.

#### 2.1.4. Experimental Procedure

Research has yet to determine the ideal duration for VR nature exposure to maximize restorative effects, but shorter sessions might be more advantageous [64]. Studies have shown considerable variation in the exposure duration, ranging from 3 s to 90 min, with a median of 2 min. In workplace settings, micro-breaks featuring green natural landscapes as brief as 40 or 90 s have been shown to restore attention and reduce tension, leading to mood and performance enhancements [65,66].

**Figure 4:**
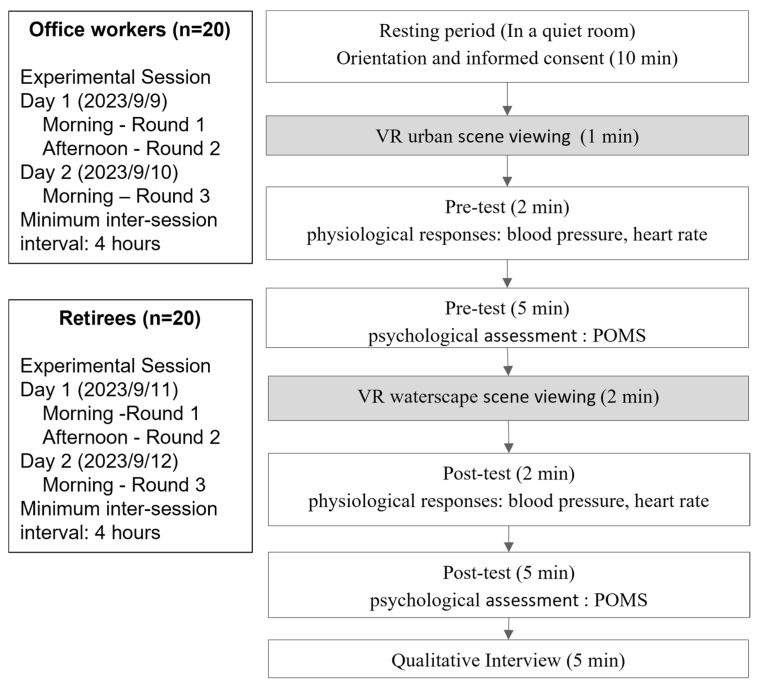
Experimental procedure.

The experimental process was segmented into six distinct phases (Figure 4): (1) participants were informed about this study and completed the consent form; (2) they observed an urban scene designed to induce stress; (3) physiological metrics such as blood pressure and heart rate were recorded, and participants filled out the Profile of Mood States (POMS), which served as the pre-test data; (4) participants then viewed a VR water scene; (5) physiological measurements were taken again, and the POMS scale was completed, serving as the post-test data; and (6) qualitative interviews were conducted to gather insights into participants’ personal experiences and landscape preferences while viewing the VR. The same tester and equipment were used for both pre- and post-test assessments for each participant to ensure consistency. Participants were free to view 360° live-action recorded images from any comfortable position and could openly share their feelings during the viewing, which were documented by the researcher.

### 2.2. Research Materials

#### 2.2.1. Participants

Previous similar studies obtained 22 samples [63] or 34 samples [57], all of which were small-sample studies. This phenomenon also reflects that although VR research has attracted more researchers’ attention in recent years, it is limited by research time, the willingness of recruiters, and the experimental process and time (filling in psychological measurement samples, measuring physiological indicators, viewing images, etc.). It is difficult for such therapeutic research to receive a large amount of survey data in a short period of time, which was one of the research limitations. As this study was exploratory, the focus was on obtaining a comparison of differences between the two groups. Subsequent research will continue to deepen this research based on this.

Participants were recruited for this study in September 2023 in Hualien, Taiwan. The twenty retirees were recruited from the senior citizen activity center in Guofeng Community, Hualien City, where they often attend classes or activities. The twenty office workers were recruited from various government agencies in Hualien, for a total of forty participants.

The participants were categorized into two groups: office workers and retirees. The rhythm of life for office workers is fixed in time and space, with wages for work and uniform vacation time. The lives of retirees are not restricted in time and space, the motivation for work is volunteering or health reasons, and there is no fixed salary.

#### 2.2.2. Video Recordings

**Figure 5:**
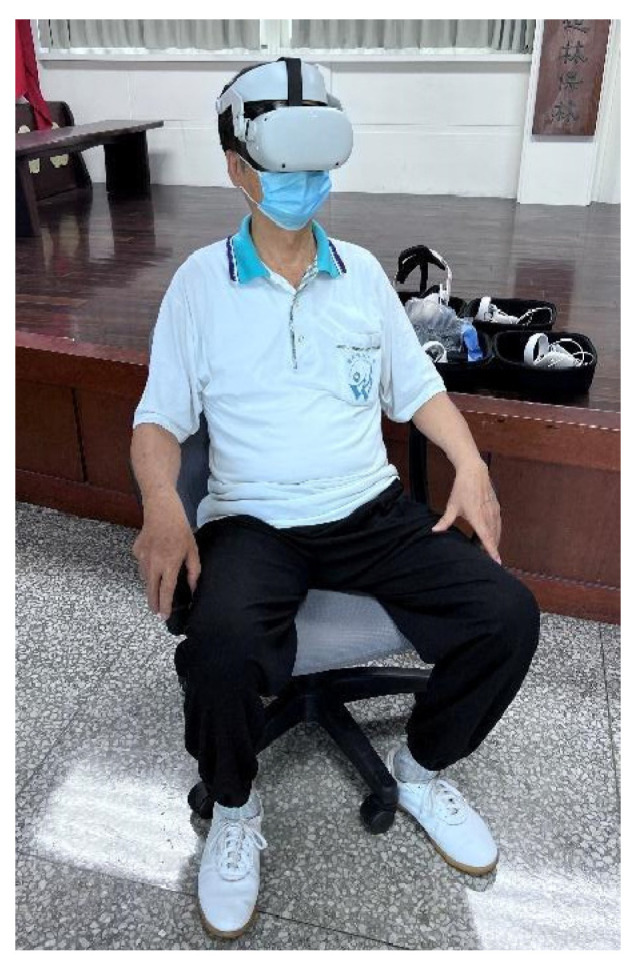
Participant wearing the Oculus Quest 2 VR device for an immersive experience of viewing a virtual waterscape.

In this study, a META Oculus Quest 2 VR headset was employed to display the recorded visuals of a 360° panoramic scene. The playback system created an immersive setting with both visual and auditory elements, enabling participants to move their heads and explore the panoramic view (Figure 5). The Insta360 X3 (Shenzhen, China) was utilized to capture 5.7 K panoramic images of a forest waterfall, a forest stream, and a beach. The forest waterfall was recorded in the Neidong National Forest Recreation Area, where water cascades down the mountainside, serving as the primary visual focus, accompanied by the sound of rushing water. Forest streams were selected from natural streams in the suburban mountains of Hualien City, where they flowed gently through the forests at a low elevation. The beach view was a gravel beach scene in the coastal environment of Hualien City (Figure 4).

### 2.3. Measurement and Analysis

#### 2.3.1. Physiological Responses

The participants’ systolic blood pressure (SBP), diastolic blood pressure (DBP), and heart rate (HR) were measured with a sphygmomanometer (HEM-7210, OMRON Corporation, distributed by OMRON Taiwan, Taipei, Taiwan).

Before the experiment began, each participant was instructed to rest quietly in a designated quiet room for at least 5 min to allow their heart rate and blood pressure to return to a stable physiological baseline. All physiological data, including systolic blood pressure and heart rate, were measured using the same automated blood pressure monitor (digital sphygmomanometer) for all participants to ensure measurement consistency. During each session, participants remained seated in a relaxed posture throughout the measurement process.

The experiment consisted of three sessions per participant, conducted across two consecutive days: two sessions (morning and afternoon) on day 1, and one session (morning) on day 2. A minimum interval of four hours was maintained between sessions to allow for sufficient physiological recovery and to minimize potential carryover effects between measurements.

#### 2.3.2. Psychological Responses

The Profile of Mood States (POMS) was employed to assess the participants’ emotional conditions. This version was derived from the abbreviated POMS created by Shacham et al. (1983) [67] and the updated Chinese POMS with altered question phrasing by Yang, Wenqi (1996) [68], Chang and Lu (2001) [69], and Hsu, P.Y. et al. (2003) [70]. The seven constructs were self-esteem (S-E) (4 items), tension–anxiety (T-A) (5 items), anger–hostility (A-H) (5 items), fatigue–inertia (F-A) (5 items), depression–dejection (D-D) (3 items), confusion–bewilderment (C-B) (4 items), and vigor–activity (V-A) (4 items). Each question was rated on a 5-point Likert scale. The Cronbach’s alpha reliability coefficient of the scale was 0.8.

The Profile of Mood States (POMS) short form was used to assess participants’ emotional states before and after the intervention. The Chinese version of the POMS, consisting of seven subscales and 30 items, has been culturally adapted and applied in previous Taiwanese studies. Specifically, Lee et al. [14] employed the revised Chinese POMS in a psychophysiological field study involving nature-based interventions in Hualien, Taiwan. The instrument demonstrated strong internal consistency, with Cronbach’s alpha values ranging from 0.82 to 0.94 across subscales. Given its demonstrated reliability and applicability in Taiwanese adult populations, including middle-aged and older adults, the scale was deemed appropriate for this study.

#### 2.3.3. Qualitative Interviews

Brief semi-structured interviews were conducted after each experiment to complement the quantitative results and gain a deeper understanding of the participants’ subjective experiences to supplement the quantitative findings. Each of the 40 participants was interviewed three times, resulting in a total of 120 interview transcripts, 1 after each session. After all the recorded interviews were converted into verbatim transcripts, the text was analyzed.

The interviews aimed to explore participants’ emotional responses to the virtual blue environments, their personal preferences regarding different landscape types, and any physical discomfort experienced during the VR exposure (e.g., dizziness or nausea). Each interview lasted approximately 2 to 3 min and was conducted immediately after the VR session to capture real-time reactions.

The qualitative data were used in a descriptive and illustrative manner to highlight notable cases and patterns observed across sessions. Participant responses were reviewed to identify representative quotes or unique experiential narratives that offered meaningful context to the quantitative results. These narrative insights were used to deepen the interpretation of emotional reactions and help explain scene-specific effects observed in the psychophysiological data.

#### 2.3.4. Data Analysis

Firstly, all participants (n = 40) were grouped into two subgroups to test the proposed hypotheses: office workers and retirees (n = 20 each). For each participant, physiological (SBP, DBP, and HR) and psychological responses (seven POMS subscales) were collected before and after viewing each of the three VR waterscape types (i.e., a forest waterfall, a forest stream, and a beach).

This study tested three hypotheses using corresponding statistical approaches. For Hypothesis 1, separate paired-sample *t*-tests were conducted within each subgroup (office workers and retirees) to examine pre–post differences in psychological (POMS subscales) and physiological (SBP, DBP, and HR) responses across the three VR waterscape conditions (a forest waterfall, a forest stream, and a beach). The results from both groups were then compared to discuss the differential effects between the populations.

For Hypotheses 2a and 2b, a series of within-group paired-sample *t*-tests were used to compare the effects of different waterscape types on each group’s psychophysiological outcomes, identifying which environment had stronger or weaker therapeutic benefits.

Hypothesis 3 was addressed via a qualitative analysis of brief post-session interviews, which collected participants’ emotional impressions and scene-specific experiences. A descriptive review of representative quotes was used to explore whether prior emotional backgrounds moderated psychological responses.

All statistical analyses were conducted using SPSS version 21.0, with the significance level set at *p* < 0.05. Both significant and non-significant results are reported to ensure analytical transparency.

## 3. Results

The valid sample included 40 individuals ranging from 29 to 81 years old, comprising 20 office employees and 20 retirees. The group was 30% male and 70% female, with 80% having completed senior high school or higher education. Additionally, 98% did not smoke, 85% did not consume alcohol, and 70% engaged in weekly exercise (Table 1). For both office workers and retirees, three separate paired-sample *t*-tests were performed per outcome variable (POMS subscales and physiological indicators) for each waterscape. All results, whether statistically significant or not, are provided in Table 2 and Table 3. Since each person needed to watch (listen to) three different virtual images (sounds) of natural water scenes and water sounds, the total number of measurements was 120.

**Analysis** **of** **Hypothesis** **1.** Independent samples revealed no significant differences in psychosocial values, such as POMS, blood pressure, and heart rate, between office workers and retirees who viewed the VR waterscapes. In contrast with the POMS test data, the baseline negative emotions of retirees were relatively low, so the degree of psychological improvement was very small, which can explain why the influence of the waterscape on them was not significant.

**Analysis** **of** **Hypothesis** **2a.** There were no significant differences in blood pressure and HR before and after viewing the forest waterfall, and there were significant differences in negative emotions such as T-A, A-H, F-I, and C-B on the POMS scale. The pre- and post-tests of viewing a forest stream showed a significant decrease in SBP and DBP, and significant differences in the POMS scales of A-H, F-I, D-D, C-B, and S-E, which indicated that the negative emotions were relieved, and the positive emotions were increased. The pre- and post-tests for viewing the beach showed a significant decrease in SBP and no significant decrease in DBP and HR, and only the T-A construct of the POMS scale showed significant differences. The virtual reality forest waterfalls, forest streams, and beach waterscapes had partially significant effects on the physiology and psychology of office workers (Table 2).

**Analysis** **of** **Hypothesis** **2b.** The pre- and post-tests of the virtual forest waterfall showed significant differences in SBP, DBP, and HR. The pre- and post-tests of the virtual forest stream showed significant decreases in SBP, but not in DBP and HR. The pre- and post-tests of the virtual beach showed significant decreases in SBP and HR, but not in DBP. There were no significant differences in the negative and positive POMS scales after viewing the three types of virtual waterscapes. The virtual reality forest waterfalls, forest streams, and beach waterscapes had partially significant effects on the physiological health promotion of the retirees (Table 3).

**Analysis** **of** **Hypothesis** **3.** The interview findings highlighted that participants’ personal emotional experiences shaped their perceptions of the landscape. Some individuals pointed out that they rarely came across waterfalls in their Hualien surroundings, which increased their enthusiasm for the waterfall feature. One participant wondered the following:

*“Where is this waterfall from? We seldom see waterfalls like this in Hualien.”*

The novelty of the waterfall view sparked positive emotions among participants, helping to mitigate their negative feelings. Regarding the beach view, since the image was taken from Hualien city’s beach, a well-known leisure spot, one participant remarked,

*“This is the beach by Nanbin Park! I often go there to walk and exercise!”*

Participants were familiar with the beach waterscape, and a comparison of the psychological benefits of the three types of waterscapes showed that the beach waterscape was the least effective in alleviating negative emotions. A retired participant shared that viewing the stream waterscape evoked childhood memories, saying the following:

*“I used to play in the stream as a child, and Grandpa taught me how to fill water with an aunt’s yam, but now I rarely visit the stream, and I miss it.”*

This participant experienced happiness due to the connection with joyful childhood memories, and the immersive virtual landscapes had a similar effect to reminiscence therapy. However, a few participants mentioned feeling scared as if they were standing in the water when viewing the forest stream and forest waterfall waterscapes.

## 4. Discussion

This study completed the experimental process according to the research design and obtained data analysis. Three main aspects will be explored in this section, namely, the effect of waterscape types on health benefits, the therapeutic potential of VR waterscapes, and research limitations.

### 4.1. Effects of VR Waterscape Types on Health Benefits

The findings suggest that for office workers, exposure to VR waterscapes significantly alleviated negative emotions such as tension, anger, fatigue, and confusion. Notably, the forest stream environment demonstrated the broadest psychological benefits, being the only scene that simultaneously enhanced positive affect—particularly self-esteem—and reduced depressive mood. Physiologically, both streams and beach landscapes led to a marked reduction in systolic blood pressure. The confirmation of the significant physiological and psychological health benefits of VR waterscapes echoes the restorative potential of blue spaces [21] and supports the therapeutic effectiveness of virtual natural waterscapes [30]. Various waterscapes, including forest waterfalls, forest streams, and beach water, provide distinct emotional and physiological benefits. Considering water movement, ocean tides are predictable, the sea remains tranquil, streams are clear and gentle, and waterfalls are grand, each evoking different sensations in people. This study points out that in the future, in-depth research can be conducted on the characteristics and psychological effects of waterscapes to facilitate the application of healing landscapes [17].

In our research, we discovered that the virtual water feature had a less pronounced psychological impact on retirees. Examining the socioeconomic backgrounds of the participants, we noted that they were all community volunteers. On-site interviews revealed their long-term involvement in community services and their generally active lifestyles. Some participants expressed that they did not experience negative emotions and felt they led happy, stress-free lives, suggesting that active community involvement among older adults enhances their psychological health and well-being [17]. Studies indicate that retirees’ active social engagement boosts their mental health and well-being [71,72], resulting in a more positive emotional baseline and reduced daily stress. The POMS pre-test scores showed that retirees had lower levels of negative emotions and higher levels of positive emotions, which might explain the minimal psychological improvement observed. However, different types of waterscapes all provided physiological benefits, particularly in lowering systolic blood pressure and heart rate. This finding aligns with previous studies indicating that exposure to real natural environments, including blue spaces, can reduce heart rate and blood pressure [11]. This study reveals that the psychological benefits of VR waterscapes are influenced by demographic characteristics, underscoring the need to tailor natural environments with specific therapeutic attributes to different demographic groups in the context of health and healing tourism, suggesting a need for further exploration in the future.

Brief exposure to natural settings has been found to boost mood [73,74], and this research highlighted that short-term exposure to VR dynamic waterscapes, featuring audiovisual stimuli, can offer psychological and physiological health benefits. These advantages include a reduction in heart rate, lower blood pressure, and a decrease in negative emotions, which are consistent with Ulrich’s stress recovery theory (SRT) [75]. Additionally, during our study, we observed that participants engaged with the VR dynamic 360° water scene by actively moving their heads to explore and observe with curiosity. The immersive aspect of VR encourages users to interact with the environment rather than just passively observe it. Active engagement and observation within the environment are essential for the recovery experience, and a higher degree of immersion amplifies these benefits [31,76].

### 4.2. The Therapeutic Potential of VR Waterscapes

This study’s interview results revealed that the restorative effects of VR waterscapes are shaped by individuals’ emotional experiences. Different emotional responses arise from people’s varied feelings toward specific water features. For example, the landscape’s novelty in this study was found to enhance positive emotions. Participants’ discomfort and fear when near a water body highlight the role of personal environmental perceptions, aligning with previous findings that environments perceived as safe are more restorative [77]. Although research on healing blue spaces often favors beach and marine environments [28], this study identified that the beach water feature, despite its physiological benefits, was the least effective in reducing negative emotions. This could be attributed to the landscape’s familiarity as a routine daily scene, which offered less novelty and exploration. Attention restoration theory suggests that restorative landscapes should provide a sense of relaxing attraction and separation from daily stress. This underscores the importance of considering individual differences and environmental perceptions when using VR to create restorative natural environments in the future [32].

In this study, a retiree reflected on childhood water activities while watching a stream, suggesting that immersive virtual environments can trigger autobiographical memories. Reminiscence therapy, which involves recalling past experiences, can improve the emotional well-being of older adults, including those with dementia [78]. VR is especially advantageous in reminiscence therapy as it provides a more engaging and realistic setting. Further investigation into VR reminiscence therapy in senior care and senior health tourism is justified, as it offers a more captivating and authentic environment, thereby effectively enhancing emotional, cognitive, and social aspects [79,80].

In this study, the urban environment of Ximending in Taipei did not consistently evoke negative emotions as a stressor. Some participants linked the area with shopping excursions, which brought them joy. One participant expressed, “This movie was filmed in Ximending, right? Seeing this place makes me so happy; I cannot stop thinking about shopping”, which diminished its effectiveness as a stress trigger by associating it with a pleasurable shopping experience. In addition to the VR sensory experience being more restricted than real-life settings due to the absence of tactile or olfactory signals [81], another reason could be that the participants were residents of Hualien and were not accustomed to the stress of a bustling and noisy city environment. As a result, viewing the urban scenery briefly was perceived as novel rather than stressful. This indicates that an individual’s emotional experience significantly impacts the psychological advantages derived from landscapes.

The qualitative interviews indicated that individuals of all ages exhibit high acceptance and low discomfort toward VR. This aligns with Alanazi et al.’s findings (2023) [82], which demonstrated that VR interventions are highly feasible, widely accepted, and cause little discomfort among older adults, thereby supporting VR’s applicability across diverse groups.

### 4.3. Study Limitations

This study was a small-sample, exploratory investigation that, despite its limited size (n = 40), offers valuable preliminary insights into the psychophysiological effects of immersive virtual blue spaces across different lifestyle groups. The findings suggest that specific environments, such as forest streams, may produce stronger emotional benefits and that group-specific tendencies can inform future digital health intervention strategies.

However, several limitations should be noted. Firstly, the participant pool was geographically limited to Hualien, Taiwan, which may restrict the generalizability of the findings to broader or more urbanized populations. Secondly, the modest sample size and subgroup distribution reduced the statistical power. Thirdly, this study assessed only short-term responses to single-session exposures to three types of waterscapes; thus, the long-term effects and sustained benefits remain unclear. Future research should consider longitudinal designs, more diverse sampling, and repeated VR exposures to better evaluate efficacy and applicability over time.

## 5. Conclusions

This study aimed to explore the therapeutic potential of blue virtual reality spaces by using two groups with vastly different lifestyles as experimental subjects. After testing the research hypothesis using experimental data from 40 samples, it was found that (1) virtual reality waterscapes had a significant effect on the physical and mental therapy of office workers, especially waterfalls and streams. (2) However, the benefits of waterscapes for retirees were not significant. The reason for the insignificant effect was not that the theme of the blue space was invalid; rather, the POMS test showed that their baseline negative emotions were low, so the degree of psychological improvement was minimal. (3) The response of retirees to urban scenes as a source of stress induction was also not significant, which can be explained by the qualitative interview text. The main reason was that Hualien has a long coastline and many water features in the forest, so blue spaces are daily landscapes, and the novelty of urban scenes strengthens positive emotions. Overall, the therapeutic potential of VR waterscapes is largely influenced by personal experience and emotions. Individual differences must be the primary consideration when using VR as a therapy medium. However, digital devices are not widely used among the elderly population, especially in areas with small populations, where health problems are more likely to be exacerbated by the digital divide. Nonetheless, the demand for digital healthcare is a future trend, and its importance must be demonstrated via research that combines social and academic value.

## Figures and Tables

**Table 1:** Sociodemographic information of the participants (n = 40).

Gender n/%		Age n/%		Occupation n/%	
Male	12/30%	≦30	1/2.5%	Business	1/2.5%
Female	28/70%	31–40	6/15%	Government employee	14/35%
Education Level		41–50	8/20%	Others	5/12.5%
Primary level or below	5/12.5%	51–60	4/10%	Retired	20/50%
Lower secondary level	3/7.5%	61–70	5/12.5%		
Upper secondary level	13/32.5%	71–80	14/35%		
Bachelor’s degree	12/30%	≥81	2/5%		
Master’s degree or above	7/17.5%				
Smoking Habits n/%		Drinking Habits n/%		Exercise Habits n/%	
No	39/98%	No	34/85%	No	12/30%
Yes	1/3%	Yes	6/15%	Yes	28/70%

**Table 2:** The biopsychological effects of VR forest waterfall, forest stream, and beach waterscapes on office workers.

Waterscape	Variables	Pre-Test	Post-Test	t-Value	*p*-Value
Forest waterfall	T-A	2.50 ± 0.83	2.17 ± 0.74	−4.714	0.000 ***
	A-H	2.51 ± 0.72	2.21 ± 0.65	−3.943	0.001 **
	F-I	2.62 ± 0.91	2.25 ± 0.71	−3.832	0.001 **
	D-D	2.06 ± 0.74	2.02 ± 0.55	−0.459	0.651
	C-B	2.50 ± 0.79	2.28 ± 0.78	−2.714	0.014 *
	V-A	3.18 ± 0.78	3.43 ± 0.74	1.969	0.064
	S-E	3.41 ± 0.55	3.51 ± 0.61	1.165	0.258
	SBP	124.90 ± 17.21	126.20 ± 18.36	0.81	0.427
	DBP	79.10 ± 11.69	78.75 ± 11.04	−0.40	0.692
	HR	75.10 ± 14.13	74.60 ± 13.57	−0.72	0.478
Forest stream	T-A	2.29 ± 0.88	2.17 ± 0.79	−1.878	0.076
	A-H	2.39 ± 0.74	2.18 ± 0.71	−2.987	0.008 *
	F-I	2.42 ± 0.86	2.23 ± 0.78	−2.263	0.036 *
	D-D	2.27 ± 0.74	2.07 ± 0.62	−2.349	0.030 *
	C-B	2.44 ± 0.80	2.23 ± 0.70	−2.540	0.020 *
	V-A	3.20 ± 0.87	3.33 ± 0.80	1.522	0.144
	S-E	3.35 ± 0.72	3.44 ± 0.70	2.101	0.049 *
	SBP	123.55 ± 14.57	119.65 ± 14.40	−3.15	0.005 **
	DBP	78.40 ± 10.90	76.15 ± 9.78	−2.70	0.014 *
	HR	74.70 ± 9.78	74.75 ± 9.35	0.09	0.929
Beach	T-A	2.38 ± 0.84	2.20 ± 0.84	−2.44	0.025 *
	A-H	2.35 ± 0.71	2.22 ± 0.73	−1.90	0.073
	F-I	2.40 ± 0.83	2.33 ± 0.85	−0.78	0.445
	D-D	2.17 ± 0.68	2.13 ± 0.70	−0.38	0.705
	C-B	2.41 ± 0.80	2.30 ± 0.78	−1.63	0.119
	V-A	3.29 ± 0.80	3.41 ± 0.82	1.14	0.268
	S-E	3.41 ± 0.68	3.46 ± 0.76	0.66	0.519
	SBP	127.25 ± 18.48	120.15 ± 15.93	−3.60	0.002 **
	DBP	79.95 ± 12.48	75.85 ± 11.26	−1.97	0.063
	HR	77.15 ± 10.21	75.50 ± 9.59	−1.63	0.120

Note: *—*p* < 0.05; **—*p* < 0.01; ***—*p* < 0.001.

**Table 3:** The biopsychological effects of VR forest waterfall, forest stream, and beach waterscapes on retirees.

Waterscape	Variables	Pre-Test	Post-Test	t-Value	*p*-Value
Forest waterfall	T-A	2.37 ± 0.82	2.23 ± 0.81	−1.629	0.120
	A-H	2.08 ± 0.54	2.00 ± 0.59	−1.322	0.202
	F-I	2.18 ± 0.72	2.10 ± 0.74	−1.094	0.288
	D-D	1.98 ± 0.63	1.93 ± 0.64	−0.719	0.481
	C-B	2.25 ± 0.54	2.21 ± 0.61	−0.616	0.545
	V-A	3.60 ± 0.64	3.66 ± 0.80	0.539	0.596
	S-E	3.75 ± 0.55	3.91 ± 0.63	1.530	0.142
	SBP	140.85 ± 19.90	135.55 ± 18.38	−2.59	0.018 *
	DBP	79.75 ± 13.08	76.80 ± 12.21	−2.15	0.044 *
	HR	77.75 ± 10.95	74.55 ± 9.75	−4.21	0.000 ***
Forest stream	T-A	2.19 ± 0.85	2.18 ± 0.93	−0.203	0.841
	A-H	2.11 ± 0.61	2.04 ± 0.58	−1.324	0.201
	F-I	2.15 ± 0.75	2.10 ± 0.79	−1.157	0.262
	D-D	2.00 ± 0.54	2.07 ± 0.65	0.809	0.428
	C-B	2.19 ± 0.68	2.20 ± 0.74	0.224	0.825
	V-A	3.60 ± 0.73	3.54 ± 0.82	−0.721	0.480
	S-E	3.88 ± 0.52	3.86 ± 0.56	−0.271	0.789
	SBP	136.65 ± 16.71	130.20 ± 16.99	−3.84	0.001 **
	DBP	75.30 ± 11.22	74.40 ± 11.92	−0.64	0.527
	HR	77.00 ± 10.82	76.60 ± 9.92	−0.64	0.530
Beach	T-A	2.10 ± 1.00	2.14 ± 0.84	0.328	0.746
	A-H	1.91 ± 0.77	2.03 ± 0.66	1.189	0.249
	F-I	2.00 ± 0.90	2.05 ± 0.75	0.461	0.650
	D-D	1.95 ± 0.83	1.97 ± 0.64	0.131	0.897
	C-B	2.11 ± 0.85	2.13 ± 0.70	0.107	0.916
	V-A	3.41 ± 1.07	3.60 ± 0.84	0.936	0.361
	S-E	3.66 ± 1.02	3.79 ± 0.67	0.567	0.577
	SBP	139.05 ± 20.44	133.05 ± 21.97	−0.99	0.022 *
	DBP	75.30 ± 11.62	74.85 ± 10.53	2.21	0.727
	HR	79.45 ± 14.41	77.05 ± 12.76	−0.94	0.003 **

Note: *—*p* < 0.05; **—*p* < 0.01; ***—*p* < 0.001.

## Data Availability

The raw data supporting the conclusions of this article will be made available by the authors on request.
